# From a Criminal to a Human-Rights Issue: Re-Imagining Policy Solutions to Homelessness

**DOI:** 10.1177/15271544231176255

**Published:** 2023-06-02

**Authors:** Tasneem Owadally, Quinn Grundy

**Affiliations:** 1 Lawrence S. Bloomberg Faculty of Nursing, 7938 University of Toronto, Toronto, Canada

**Keywords:** housing, homelessness, criminalization, encampments, human rights, public policy, nursing advocacy

## Abstract

Criminalizing homelessness is ineffective, costly, and immoral; yet it remains a dominant feature in the management of this global social issue. There has been little analysis investigating why punitive homeless policies have remained popular despite their ineffectiveness. In applying Bacchi's What's the Problem Represented to Be (WPR) framework to a Canadian encampment bylaw, our analysis demonstrated that public policies criminalizing homelessness continue to prevail because homelessness is fundamentally understood as a problem of deviant, criminal individual behavior. We argue that reframing understandings of homelessness from one of criminality to a human rights issue gives way to more dignified, just, and effective solutions, such as the Housing First Model. We suggest that community health nurses can serve a key role in disrupting these criminalizing discourses across domains of policy, research, and practice by advocating for holistic, rights-based, and equity-oriented policy solutions related to homelessness.

## Introduction

Homelessness is a persistent and growing global social problem which has been exacerbated by the coronavirus disease 2019 (COVID-19) pandemic, widening longstanding population inequities in housing affordability, employment, and income (Olson & Pauly, 2021). For example, the 2022 annual report from the United States (US) Department of Housing and Urban Development (HUD, 2022) estimated that approximately 582,500 Americans or 0.18% of the total population experience homelessness on a given night. A higher percentage was reported in the 2022 Canadian point-in-time prevalence, where an estimated 1% of the total population (i.e., approximately 35,000 individuals) were found to be homeless on a nightly basis (Statistics Canada, 2022). What it means to be homeless varies considerably across countries; however, homelessness is broadly defined as “the situation of an individual, family or community without stable, safe, permanent, appropriate housing, or the immediate prospect, means and ability of acquiring it” (Canadian Observatory on Homelessness, 2021, p. 1).

More specifically, the growth in housing insecurity is reflected in the rise in encampment sites, noted in countries across the world (Aldanas, 2020; National Homelessness Law Centre [NHLC], 2017). A homeless encampment refers to a temporary structure such as a tent or shack used for shelter in an area that is not intended for habitation (Rankin, 2019). For instance, the US has seen an exponential growth in outdoor homelessness, as evidenced by a 1,342% increase in documented encampment sites between 2007 and 2017 (NHLC, 2017). Similarly, this phenomenon can be seen in Europe, where the Federation of National Organizations Working with the Homeless (FEANSTA) (FEANTSA, 2017), a prominent advocacy group seeking to end homelessness, reported that street poverty is on the rise in most of Europe.

As a result, municipal governments have been facing a growing pressure from multiple policy stakeholders to respond to the increase in visible homelessness in their cities, making this issue a highly politicized matter with polarized plans of action (Cohen et al., 2019). Punitive laws, in the form of encampment evictions, have unfortunately been the dominant approach in the management of outdoor homelessness in North America and Europe (Aldanas, 2020; Cohen et al., 2019; NHLC, 2017). These punitive laws have been highly detrimental to the health and well-being of homeless individuals as they have turned basic life-sustaining activities, such as sleeping and sheltering into punishable offences, thus, criminalizing their very existence in public spaces (Rankin, 2019).

Homelessness should be of concern not only to policymakers but also to nurses because housing is a fundamental social determinant of health (Raphael et al., 2020). As a result, unsafe, unaffordable, insecure, or the absence of housing puts individuals at risk for a host of health problems (Raphael et al., 2020). Failure to address homelessness would therefore be a direct violation of the nursing profession's responsibility to uphold the principles of justice and equity in the distribution of the social determinants of health (International Council of Nurses, 2021).

Nurses, across domains of nursing practice, are also expertly positioned to contribute to the identification, analysis, and development of the policy conditions and structures that can counter homelessness. Congruent with a critical approach to policy analysis (Bacchi, 2009), a nursing lens allows for situating a social issue holistically within its broader socioeconomic and political contexts and attending to intersecting forms of oppression, power, and privilege (Disch, 2020). Additionally, a nursing lens within policy analysis allows for incorporation of unique insights into the lived effects of policy solutions gained from nurses’ interactions with and knowledge of the experiences of diverse populations (Disch, 2020).

Currently, many policy strategies to address visible homelessness are inhumane, ineffective, and costly (Rankin, 2019). For instance, police officers are often called upon to enforce municipal bylaws criminalizing encampments, giving them the legal power to forcibly evict homeless individuals from public spaces (Rankin, 2019). These clear-outs provide the public with a temporary illusion that homelessness is being ‘solved; however, they simply force encampment residents to build new camping grounds in less visible spaces or go to overcrowded shelters (Loriggio, 2021; Rankin, 2019). This approach to managing encampments is not only counterproductive, but also highly expensive, costing cities between 1,672 and 6,208 US dollars per unsheltered individual annually (Dunton et al., 2020).

Ineffective strategies to address homelessness result in significant indirect cost, as homeless individuals are heavy users of publicly funded services such as the health care system (Rankin, 2019). The COVID-19 pandemic has highlighted the inextricable link between adequate housing and health, as unsheltered individuals, who were unable to practice recommended public health measures due to their precarious living conditions, became vulnerable to infection and thus, disproportionately represented in COVID-19-related hospitalizations and fatalities (McGillivray, 2021). Homelessness is therefore an ethical, legal, social, and health crisis, in need of long-term, coordinated, and integrated solutions.

Identifying effective public policy solutions requires a clear understanding of how homelessness is represented as a problem within proposed policy interventions. Drawing on Bacchi's (2009) What's the problem represented to be (WPR) critical approach to policy analysis and the authors’ nursing perspectives, this paper analyzes the problem presentations contained within the City of Toronto (2021) bylaw banning camping in city parks to better understand the problem representation of homelessness within municipal policies that criminalize encampments. Since the criminalization of homelessness is a dominant approach in various countries (Aldanas, 2020; NHLC, 2017), analysis of this Canadian case study carries important implications for shared understanding of the problematic ways in which homelessness is constructed as a public policy problem and allows for the opportunity to reframe these issues and further the collective development of effective policy strategies.

In the following analysis, we examine how the criminalization of encampments contributes to a problem representation of homelessness that fails to address its root causes and instead, reinforces deep-seated negative stereotypes, leading to further marginalization of people experiencing homelessness. After discussing the emergence and management of homelessness as a public policy problem in Canada, we present an overview of the WPR approach and its application to the City of Toronto's (2021) encampment bylaw. We show how policy choices to criminalize encampments have implicitly problematized homelessness as a societal burden, defined by personal failure and individual blame, thus normalizing the use of law enforcement towards homeless individuals (Rankin, 2019). We conclude with a call to embed rights-based problem representations for homelessness within the development of social policies to generate more effective, health-promoting policy solutions at the local, national, and international level.

## Historical Origins of Homelessness as a Public Policy Problem

The recognition of homelessness, as a social issue in need of policy intervention, is relatively new, arising in the 1980s when widespread welfare cuts resulted in developed nations such as Canada seeing an alarming rise in individuals becoming unhoused (Hulchanski, 2009). By the turn of the twenty-first century, the health service community also saw a shift in the understanding of health, that evolved from a predominantly biomedical model to a more holistic approach that considered the socioeconomic context that influences health outcomes (World Health Organization [WHO], 2008). This shift was reflected in the 2008 WHO Commission on Social Determinants of Health, aimed at drawing attention to the systemic factors leading to health inequity. The WHO Commission report repeatedly emphasized affordable, quality housing as a fundamental requirement for healthy living (WHO, 2008). Homelessness was shown to be strongly associated with poor health outcomes, as evidenced by the high prevalence of chronic diseases, mental health disorders, and communicable illnesses among this vulnerable population compared to housed individuals (Rankin, 2019). Homelessness, therefore, became recognized in the health care field as a complex social problem stemming from intersecting structural factors of poverty, lack of affordable housing, and unemployment, compounded by personal circumstances such as illnesses, addictions, and social relations (WHO, 2008). Unfortunately, this progressive understanding of homelessness, as rooted in underlying social and structural conditions, has not been met with effective strategies to address homelessness (Rankin, 2019). Proposed evidence-based interventions for the management of homelessness have encountered significant resistance under neoliberal governments that do not prioritize public spending towards welfare programs and instead, opt for politically expedient, short-term solutions that fail to address the complexity of this social issue (Nelson et al., 2021).

However, recent policies that in contrast, embed a human-rights approach, suggest that these issues can be addressed ethically and sustainably. Finland has demonstrated how a welfare state can successfully address housing insecurity, as it has become the only nation in the European Union where homelessness is declining (Juhila et al., 2022). In alignment with its national mandate to eradicate street poverty by 2027, Finland allocated sustained funding to social protection systems, such as the evidence-based Housing First model (Juhila et al., 2022). Guided by the core Housing First principle that housing is the first fundamental step to emerge from homelessness, the Finnish federal government supported its homeless population in re-integrating into society by providing them with timely access to permanent housing (Juhila et al., 2022). These robust welfare programs have allowed the country to protect its vulnerable population from economic downturns, as illustrated in the COVID-19 pandemic, during which homelessness has continued to decrease from 16,000 in 1989 to around 4,000 in 2020, representing only 0.08% of the population (Boone et al., 2021). At a city level, the US city of Boston also demonstrated how a holistic, person-centered, and coordinated multisectoral approach, can help to re-house homeless individuals without criminalization (United States Interagency Council on Homelessness, 2022). Nurses, in point-of-care, health systems management and leadership, and policy roles are expertly positioned to plan, execute, evaluate, and advocate for these models given the congruence with nursing models of care (Disch, 2020).

## Emergence and Management of Homelessness: A Canadian Case Study

The state of homelessness in Canada has been highly susceptible to changes in the sociopolitical climate. Prior to the end of the second world war in 1945, federal governmental involvement was relatively limited in the Canadian housing market (Hulchanski, 2009). A shift occurred, starting in the 1950s, when elected officials became deeply invested in improving Canadians’ postwar standard of living and thus, “created a functioning mortgage system with government mortgage insurances, built social housing, and subsidized private-sector rental units” (Hulchanski, 2009, p. 3). This period also saw the establishment of welfare programs such as universal health care, the national pension plan and unemployment insurance (Hulchanski, 2009). These social safety nets meant that Canadians were provided the necessary federal assistance to prevent the extreme poverty that can lead to homelessness (Hulchanski, 2009).

However, in the early 1980s, a newly elected Conservative government under the leadership of Brian Mulroney, embarked on major cutbacks to social assistance programs (Greene, 2014). For example, in 1984, the national subsidized housing program sustained a substantial 217.8 million dollar cut in funding (Greene, 2014). By 1993, the federal government ceased all spending on the construction of new social housing units and relegated this task to provinces, absolving itself from the responsibility (Hulchanski, 2009). With the loss of federal subsidy, the housing sector became increasingly disincentivized to build affordable social units and, instead invested in the more profitable private-market real estate (Greene, 2014).

During this time, Toronto, Canada's largest city, saw a stark rise in individuals using emergency shelter services from fewer than 2,000 people in 1982 to 22,000 by 1988, making street poverty increasingly visible (Greene, 2014). As Toronto was gaining status as the financial and business capital of Canada, public displays of homelessness were seen as detrimental to the City's overall image of prosperity (Greene, 2014). This resulted in the use of punitive laws to reduce the visibility of homelessness in public spaces through the adoption of anti-begging laws such as the 1999 Ontario Safe Streets Act allowing police officers to issue fines to individuals soliciting money in public spaces (Gaetz, 2013).

To this date, these discriminatory practices overtly continue, as evidenced by the violent evictions that took place in the City of Toronto's parks in the summer of 2021. These evictions were permitted due to revisions to the bylaw under chapter 608, section 13 of the Toronto Municipal Code which stipulates that “unless authorized by permit, no person shall dwell, camp or lodge in a park” (City of Toronto, 2021, p. 14). These revisions were upheld despite appeals from homeless advocacy groups for a court injunction which would have resulted in a province-wide eviction moratorium during the pandemic (Gibson, 2020). Instead, the Ontario Superior Court of Justice, the highest provincial court level to oversee criminal offenses, found that the City of Toronto had taken sufficient steps to justify the clear-outs by establishing the Pathway Inside program – a city-run initiative to provide encampment residents with “safe” alternative indoor options in shelters or hotels (Gibson, 2020). However, this court ruling failed to account for the deplorable conditions of these indoor spaces as poor infection control measures rendered the shelter system extremely unsafe (McGillivray, 2021). At a time where transmission was rampant and vaccinations were scarce, shelter-users became disproportionately represented in COVID-19 related morbidity and mortality (McGillivray, 2021).

In addition, the revised bylaw gave the City of Toronto the legal power to evict encampment residents, resulting in multiple clashes between the police and this homeless population. Armed in full riot gear, enforcement officials dispersed the crowd of supporters using batons and pepper sprays and dismantled the encampment sites (Loriggio, 2021). Given the ongoing and rising rates of homelessness and poor health outcomes among homeless populations in Toronto (Statistics Canada, 2022), this bylaw is far from being effective and instead, represents a failed public policy in addressing homelessness as a health and health equity issue. However, to understand why an approach to the problem consistently favors criminalization and violence toward people experiencing homelessness, it is first necessary to understand how the problem of homelessness is constructed implicitly and explicitly within the policy arena.

## Understanding What the Problem is Represented to Be

The WPR approach serves as a tool to critically deconstruct a policy and examine how a problem is represented within its proposed policy intervention (Bacchi, 2009). The WPR approach rests on the assumption that problems are socially constructed, that is, society's creation of a shared understanding of ideas to make sense of reality (Bacchi, 2009). Policies also play a role in understandings of a particular set of conditions by implicitly defining them as a problem worthy of policy action. The choice of a policy intervention further defines a problem as a particular kind of problem and is imbued with normative assumptions about what kind of action should be taken and how the people involved should be treated (Bacchi. 2009).

The concept of problematization (the social process of collectively describing a phenomenon as a problem (Bacchi, 2009)) is useful for understanding how homelessness came to be understood as a widespread social problem requiring policy action, which occurred in Canada in the 1980s (Hulchanski, 2009). Problematization frames how an issue is understood, which is what Bacchi (2009) refers to as a problem representation. Bacchi (2009) suggests that there are various means of representing a problem but, groups in positions of authority push for certain representations to become the dominant way of thinking which, in turn dictate what solutions are proposed for the given issue.

Bacchi's WPR approach (2009) differs from traditional policy analysis, which understands policies as solutions to pre-existing, objective societal problems. Rather, policymakers are active agents in promoting certain problem representations through their choice of interventions (Bacchi, 2009). Policies are therefore value-laden and should be subject to critical scrutiny to understand how they frame a problem. The WPR approach therefore provides an equity-oriented theoretical lens, which when combined with a nursing perspective that provides insight into the lived effects (Disch, 2020), allow for identification of the normative assumptions underlying punitive homeless policies, so that alternative problem definitions can be identified that will give way to more humane, health-promoting, and effective solutions.

To critically analyze a given policy, Bacchi (2009) provides the reader with a structured approach using six interrelated questions. These questions and their aims are summarized in Table 1. In the next sections, we will apply the WPR framework to critically analyze the City of Toronto's encampment bylaw and demonstrate how policy decisions to criminalize encampments have profound implications on how homelessness is understood as a societal problem. Our analysis will attempt to disrupt this problematic representation of homelessness, as rooted in criminality, and instead, we will advocate for a human-rights approach to the management of this social problem.

**Table 1.** Summary of What's the Problem Represented to Be (WPR) Questions and Aims

WPR Questions	Aims
1. **What is the “problem” represented to be?**	To highlight the dominant problem representation in a given policy.
2. **What presuppositions or assumptions underlie this representation of the problem?**	To uncover entrenched societal values and assumptions that have led to our understanding of a problem.
3. **How has this representation of the “problem” come about?**	To examine the genealogy of the implied problem representation.
4. **What is left unproblematic in this problem representation? Can the problem be thought differently?**	To interrogate how a particular problem representation favors certain problematization while precluding others.
5. **What effects are produced by this representation of the problem?**	To unpack the effects of the problem representation, especially how it may harm certain groups in society.
6. **How/ where is this representation of the problem produced, disseminated, and defended? How could it be disrupted?**	To examine the means through which a particular problem representation comes to dominate the societal discourse.

*Note.* Adapted from “Introducing a “what's the problem represented to be? approach to policy analysis,” by C. L. Bacchi, in *Analysing policy: What's the problem represented to be?* (p. 1–24), 2009, Frenchs Forest, N.S.W: Pearson Education.

### Criminalizing Homelessness- Identifying the Dominant Problem Representation

The City of Toronto's camping ordinance, stipulating that “unless authorized by permit, no person shall dwell, camp or lodge in a park” (City of Toronto, 2021, p. 14) is targeted at controlling homeless individuals within public spaces. Hence, it implicitly defines the conditions of homelessness as a particular kind of problem for which the encampment bylaw serves as a solution (Bacchi, 2009). In identifying the problem definition constructed by this policy, the encampment bylaw proposes to use law enforcement as a strategy to restrict the use of public spaces and to manage outdoor homelessness through the threat of arrests, fines, and incarceration for those who fail to comply (City of Toronto, 2021). Within this proposed intervention, homelessness is thus framed as a legal problem within the realm of the criminal justice system.

### Uncovering Public Assumptions Related to Homelessness

In analyzing a given policy, Bacchi (2009) asks readers to consider the taken-for-granted beliefs underpinning a problem representation. In the case of homelessness, community support for the criminalization of encampments often rests on the assumption that homeless individuals pose a threat to public safety (Gaetz, 2013; Olson & Pauly, 2021). Rankin (2019) addresses this myth by demonstrating that homeless individuals are no more likely to engage in criminal activities than a housed person, except for infringement of laws that punish people for living outdoors, such as trespassing or loitering. Contrary to this, Rankin (2019) suggests that unsheltered people are at an increased risk of being victims of violent crimes. Unfortunately, this public misconception of homeless individuals as “dangerous” and “delinquent” remains prevalent and is used to justify punitive laws against this marginalized group to maintain safe neighborhoods (Gaetz, 2013). In addition, encampments are often displaced because visible homelessness is believed to be detrimental to the overall image of a city's prosperity (Speer, 2019). Speer (2019) describes how public displays of urban poverty in the form of encampment sites, are seen as aesthetically unpleasant, causing widespread disgust, fear, and avoidance among the public. These evoked emotions are believed to affect the retention and expansion of economic activities; thus, city officials often face political pressure from powerful and positively constructed policy stakeholders such as businesses to clear out encampments (Speer, 2019). Public policy choices are therefore heavily influenced by groups with political leverage (Schneider & Ingram, 1993); as a result, elected officials become invested in the displacement of encampments to win over business corporations who possess significant voting power.

Homelessness is also often criminalized because of the common belief that individuals become homeless because of poor choices and personal failures (Olson & Pauly, 2021; Rankin, 2019). Another stereotype closely linked is the idea that homeless people are lazy and abuse the welfare system paid for by hardworking taxpayers (Rankin, 2019). These public perceptions are rooted in neoliberal values that are used to discourage governmental funding for social assistance programs and instead, stress the importance of individual responsibility (Rankin, 2019). These prejudices reduce public compassion towards homelessness and are often used to justify dismantling encampment sites (Olson & Pauly, 2021). Homelessness is therefore plagued by negative discourses of criminality and individual blame that are used to rationalize the eviction of encampment residents (Olson & Pauly, 2021).

Stigmatizing attitudes towards individuals experiencing homelessness are pervasive, including within the nursing profession (Zeien et al., 2021). In their scoping review, Groves et al. (2021) found that nurses share the general public's negative perceptions against marginalized social groups, such as the homeless population, impacting the quality of care provided. These stigmatized communities were found to receive inferior healthcare treatment with shorter nurse-patient interaction time, poorer communication, and suboptimal pain management (Groves et al., 2021). Zeien et al. (2021) describe how these discriminatory healthcare encounters act as significant barriers in accessing care as unsheltered individuals tend to delay seeking medical attention, presenting late in their disease process with acute problems that could have been prevented with the proactive utilization of primary care services. Hence, negative discourses of criminality reinforced in anti-camping ordinances, have diverse and profound stigmatizing impacts on the homeless population, with direct lived effects on their health and well-being.

### Failure to Problematize Homelessness as a Human Rights Issue

In the WPR framework, Bacchi (2009) also draws our attention to consider how a problem representation limits our ability to see an issue differently. The criminalization of encampments fails to problematize that the root causes of homelessness lie in governmental failures to adequately support low-income Canadians and instead, shifts the blame for systemic deficiencies onto homeless individuals (Olson & Pauly, 2021). A focus on individual blame limits one's ability to recognize that the underlying causes of homelessness, such as lack of access to affordable housing and unemployment, are issues that rest beyond an individual's control (O’Grady et al., 2011). It also ignores the fact that people often experience homelessness because of unforeseen personal circumstances such as physical illnesses, mental health disorders, or abuse, which are tragedies that can affect anyone in society (Olson & Pauly, 2021). Understanding the root causes of homelessness, therefore, gives way to a more humane approach in responding to this social problem. It allows us to redefine the problem representation of homelessness from one of criminality to a human rights issue. Adopting a human-rights perspective shifts our strategies to focus on homeless individuals’ social determinants of health to ensure equitable access to adequate housing (Olson & Pauly, 2021).

### Stigmatizing and Traumatic Effects of Criminalizing Homelessness

In Bacchi's WPR framework (2009), the reader is asked to consider three types of impacts, namely the discursive, subjectification, and lived effects. Discursive effects refer to the consequences that result from being unable to think about a problem differently (Bacchi, 2009). A discursive effect of criminalizing encampments is that it prevents society from considering why encampment residents choose to live outdoors. For example, during the pandemic, homeless individuals in Toronto reported that they preferred living outside because of fear of contracting the COVID-19 virus in overcrowded shelters or hotels that were being offered through the city-run Pathway Inside program (Neufeld, 2021). Other reasons cited were due to previous negative experiences of physical violence, theft, and rape in shelters (Neufeld, 2021). These fears are legitimate given the findings that showed that violent incidents in Toronto's city-run shelters have increased by more than 200% from 120 incidents per month in 2016 to 368 in early 2021 (Neufeld, 2021). Hence, making encampments illegal limits one's ability to interrogate why shelters are so unsafe which prevents us from recognizing that homeless individuals choose to live outdoors because they are left with no safer alternatives.

In addition, the criminalization of homelessness can have profound subjectification effects on the homeless population. Subjectification effects refer to the impacts that a problem representation has on the way people feel about themselves (Bacchi, 2009). Having a criminal record, even for minor offenses such as infringement of an anti-camping ordinance, can hinder a person's efforts to obtain employment and housing (Gaetz, 2013). In turn, these failed attempts can lead to feelings of hopelessness, despair, and self-blame (Gaetz, 2013). Hence, criminalization leads to a vicious cycle that prevents a person from emerging from homelessness and instead, results in internalized feelings of blame.

Anti-camping ordinances also have direct lived effects on homeless individuals, as evidenced by the trauma reported in Toronto's 2021 evictions. The forced displacement led to physical harm, destruction of personal belongings, and a loss of community, resulting in feelings of social isolation (Loriggio, 2021). More broadly, the criminalization of homelessness also inflicts a long-lasting psychological toll on unsheltered individuals by subjecting them to greater police surveillance (O’Grady et al., 2011). In a report on the policing of homeless youth in Toronto, 75% of respondents reported having repeated encounters with the police, with the majority being negative in nature and consisting of unsolicited identification checks, interrogations, or tickets for minor misdemeanors (O’Grady et al., 2011).

### Media as a Powerful Tool to Defend or Disrupt Societal Discourses

The media plays a key role in the construction of homeless individuals as a social group worthy of a particular policy action (Schneider & Ingram, 1993; Zufferey, 2014). According to a study by Zufferey (2014), media representations of homelessness tend to be overwhelmingly negative in nature, depicting unsheltered individuals in a vilifying manner as “criminals,” “drug addicts” or “slackers.” These portrayals serve to reinforce negative stereotypes and promote the criminalization of homelessness (Zufferey, 2014). Schneider and Ingram (1993) explain how the normative and evaluative dimensions ascribed to a social group have a powerful influence on the choice of policy intervention. As such, public officials often inflict punishment on negatively constructed social groups, such as the homeless population, knowing that the public will likely respond favourably to a harsh approach in controlling these groups that are widely viewed as deviant (Schneider & Ingram, 1993). Hence, media heavily influences the social construction of homelessness as a problem and the types of policy interventions that are deemed acceptable.

However, the media served a more positive role in the evictions that took place in Toronto by giving insight into the trauma caused by the clear-outs (Neufeld, 2021). News outlets circulated videos of the police response and provided a platform for the perspectives of encampment residents (Loriggio, 2021). These news coverages conveyed a sense of urgency into this crisis and helped to gather public support for a more humane approach to addressing homelessness in Toronto (Loriggio, 2021). The footage that emerged from the standoff between the police and supporters, therefore, served as a mean to hold enforcement officials accountable for their excessive use of power to manage the situation. Unfortunately, the City of Toronto's former Mayor, John Tory, did not respond positively to the large public outcry against the police response and instead, defended the clear-outs by describing them as “reasonable, firm, but compassionate” (Herhalt, 2021). Former Mayor John Tory's response is reflective of the broader media representation of homelessness, as a societal burden, which he utilized to legitimize the use of coercive legal sanctions as a “reasonable” approach in managing encampments.

## Implications for Nursing

This analysis offers several important implications for nurses, but also for policy analysis and development. First, we suggest that a nursing lens is highly useful in combination with critical policy analysis approaches, such as Bacchi's WPR (2009). Disch (2020) argues that the effectiveness of the equity-oriented nursing lens in analyzing and promoting effective public policy is exemplified in the countless formal and informal advocacy work that point-of-care nurses engage in within their day-to-day practice to address health disparities faced by vulnerable social groups. A prominent example is Cathy Crowe, a Canadian street nurse that has gained international recognition for her relentless commitment in fighting for the rights of homeless individuals (Cathy Crowe, n.d.). Her remarkable work as the co-founder of the 1998 Toronto Disaster Relief Committee allowed homelessness to become declared a Canadian national disaster, in need of emergency humanitarian relief (Cathy Crowe, n.d). Her inspiring contribution is indicative of the broader nursing profession's capacity to effect meaningful policy changes at the international, federal, and regional level.

Secondly, in reframing the problems that underpin current approaches to homelessness, this analysis points to existing evidence-based solutions such as the Housing First approach to tackle the rise in outdoor homelessness. Nurses and nursing advocacy groups can draw on these models to oppose the traditional “treatment first” approach that requires participants to demonstrate readiness for housing through lifestyle changes. In contrast, Housing First is guided by the principle that adequate housing is a fundamental right and is the first step necessary to successfully emerge from homelessness (Hagy, 2021). The effectiveness of this approach is supported by findings from several randomized control trials conducted in diverse socioeconomic and cultural contexts that demonstrated that the Housing First model has double the success rate than the “treatment first” approach at keeping individuals housed (Hagy, 2021). Hence, the adoption of a Housing First approach, coupled with investment in permanent supportive housing, can offer effective long-term solutions to re-housing encampment residents.

In addition, our analysis was important in highlighting that homelessness is a complex social issue; as such, it cannot be realistically solved by any singular discipline but instead, requires coordinated cross-sectoral collaboration (Abdel-Samad et al., 2021). To have a meaningful impact, nursing organizations will need to synergize advocacy efforts across and within disciplines. Abdel-Samad et al. (2021) recommend the use of multidisciplinary task forces to bring together stakeholders under a shared community agenda. This unified approach can help to centralize efforts of academics, policymakers, practitioners, and individuals with lived experiences by integrating their expertise towards the development of dignified solutions that will effectively address homelessness (Abdel-Samad et al., 2021).

In alignment with the literature, this paper also revealed that uptake for evidence-based solutions to social problems do not occur in a linear and rational manner; instead, they must compete for attention with other proposed interventions within the policymaking arena (Nelson et al., 2021). Kingdon (1995) introduces the idea of policy windows, which are critical moments where elected officials become receptive to proposed policy interventions to address a pressing problem. “Policy entrepreneurs” who are highly motivated change agents, possess the political astuteness to take advantage of these opportune moments to leverage their ideas as the ideal solution to a given problem (Kingdon, 1995). With adequate political training, we propose that nurses can act as “policy entrepreneurs” to identify these windows of political receptivity.

Despite the profession's historical involvement in social justice activism, Rasheed et al. (2020) describe that nurses do not tend to engage in policy work and often prefer remaining politically neutral. Common challenges cited in the literature related to the actualization of nurses’ political potential include a lack of time, confidence, training, and motivation (Rasheed et al., 2020). However, the impact of failed public policies during the COVID-19 pandemic has resulted in an increased number of clinical nurses becoming more politically engaged. This renewed political interest was evident in the 2022 provincial election in Ontario, Canada, which saw a record number of registered nurses running as candidates with important public health-related issues on their mandates, including affordable housing and homelessness (Payne, 2022). Future studies should explore these nurses’ experiences so that enabling structures and processes (i.e., political training courses, funding opportunities, nursing curriculum changes, mentorship) can be developed to support their political endeavors.

## Conclusion

This paper revealed that criminalization, despite its ineffectiveness, remains a prevalent feature in community responses worldwide because homelessness is fundamentally understood as a problem of criminal, deviant behavior. These negative discourses are problematic because they limit our ability to recognize that the root causes of homelessness lie in intersecting structural factors that perpetuate social inequities. To be effectively solved, homelessness needs to be redefined as a human-rights issue. Through disrupting the criminalizing discourses around homelessness, competing equity-oriented problem representations can be used to challenge punitive homeless policies, creating space for the creation of socially just welfare policies that offer a dignified approach to solving homelessness.
